# *N*-methyl-D-aspartate receptor-mediated glutamate transmission in nucleus accumbens plays a more important role than that in dorsal striatum in cognitive flexibility

**DOI:** 10.3389/fnbeh.2014.00304

**Published:** 2014-09-05

**Authors:** Xuekun Ding, Yanhua Qiao, Chengji Piao, Xigeng Zheng, Zhengkui Liu, Jing Liang

**Affiliations:** ^1^Key Laboratory of Mental Health, Institute of Psychology, Chinese Academy of SciencesBeijing, China; ^2^University of Chinese Academy of SciencesBeijing, China

**Keywords:** NMDA receptor, AMPA receptor, striatum, set-shifting, reversal learning

## Abstract

Cognitive flexibility is a critical ability for adapting to an ever-changing environment in humans and animals. Deficits in cognitive flexibility are observed in most schizophrenia patients. Previous studies reported that the medial prefrontal cortex-to-ventral striatum and orbital frontal cortex-to-dorsal striatum circuits play important roles in extra- and intra-dimensional strategy switching, respectively. However, the precise function of striatal subregions in flexible behaviors is still unclear. *N*-methyl-D-aspartate receptors (NMDARs) are major glutamate receptors in the striatum that receive glutamatergic projections from the frontal cortex. The membrane insertion of Ca^2+^-permeable α-amino-3-hydroxy-5-methyl-4-isoxazole-propionic acid receptors (AMPARs) depends on NMDAR activation and is required in learning and memory processes. In the present study, we measured set-shifting and reversal learning performance in operant chambers in rats and assessed the effects of blocking NMDARs and Ca^2+^-permeable AMPARs in striatal subregions on behavioral flexibility. The blockade of NMDARs in the nucleus accumbens (NAc) core by AP5 impaired set-shifting ability by causing a failure to modify prior learning. The suppression of NMDAR-mediated transmission in the NAc shell induced a deficit in set-shifting by disrupting the learning and maintenance of novel strategies. During reversal learning, infusions of AP5 into the NAc shell and core impaired the ability to learn and maintain new strategies. However, behavioral flexibility was not significantly affected by blocking NMDARs in the dorsal striatum. We also found that the blockade of Ca^2+^-permeable AMPARs by NASPM in any subregion of the striatum did not affect strategy switching. These findings suggest that NMDAR-mediated glutamate transmission in the NAc contributes more to cognitive execution compared with the dorsal striatum.

## Introduction

Strategy switching as a cognitive ability is critical for adapting to and surviving in an ever-changing environment. Impaired cognitive flexibility is one of the core symptoms of schizophrenia (Orellana and Slachevsky, 2013). Considerable studies in humans (Hampshire and Owen, 2006; Chamberlain et al., 2008) and animals (Ragozzino et al., 1999; Birrell and Brown, 2000) indicate that the medial prefrontal cortex (mPFC) is involved in switching attention between stimulus dimensions, whereas the orbitofrontal cortex (OFC) mediates reversal learning intradimensionally. The striatum is one of the major brain regions to which frontal cortex glutamatergic neurons project (Reynolds and Zahm, 2005; Xu and Südhof, 2013), but the role of this region in cognitive flexibility is not well-known.

The systemic blockade of the GluN2B subunit of *N*-methyl-*D*-aspartate receptors (NMDARs) selectively impairs behavioral flexibility without influencing initial reward-response association learning (Dalton et al., 2011). Subchronic treatment with the noncompetitive NMDAR antagonist ketamine reduces perseverative errors during extradimensional set-shifting but impairs intradimensional reversal learning (Floresco et al., 2009). Set-shifting ability is impaired by subchronic treatment with the NMDAR antagonist phencyclidine (Egerton et al., 2008). The phenotypes induced by these treatments closely resemble heterogeneous symptoms in schizophrenia patients (Jentsch and Roth, 1999), implying that NMDARs in the brain participate in the process of replacing old strategies with new ones. However, the precise brain region where NMDARs play a role in cognitive flexibility requires further elucidation. NMDARs in the striatum may make a large contribution to this type of flexible behavior. NMDARs are widely expressed on striatal neurons that receive glutamate inputs from the frontal cortex. However, only a few studies have suggested that NMDAR activation in the dorsomedial striatum (DMStr) and dorsolateral striatum (DLStr) differentially contribute to reversal learning (Palencia and Ragozzino, 2004, 2005). In addition to NMDARs, α-amino-3-hydroxy-5-methylisoxazole-4-propionate receptors (AMPARs) are another main type of glutamatergic receptor. AMPARs are divided into GluA2-lacking and GluA2-containing receptors, which have different calcium permeability (Plant et al., 2006; Isaac et al., 2007). Although calcium-permeable GluA2-lacking AMPARs comprise a small proportion of the normal central nervous system, their insertion into the synaptic plasma membrane occurs during the early phase of long-term potential (LTP), subsequently triggering GluA2-containing AMPAR insertion into the membrane (Jaafari et al., 2012). NMDAR activation modulates GluA1 subunit phosphorylation and dephosphorylation, which are associated with GluA2-lacking AMPAR trafficking and long-term plastic changes (Toyoda et al., 2009; Opazo et al., 2010; Ai et al., 2011). The infusion of the AMPAR general antagonist LY293558 and NMDA receptor antagonist MK-801 into the mPFC significantly increased the number of trials required to reach criterion performance in a set-shifting task (Stefani et al., 2003). In aged rats with impaired set-shifting ability, higher levels of NMDAR binding in the DMStr are correlated with poorer set-shifting performance, with no changes in AMPAR binding level (Nicolle and Baxter, 2003). Thus, an unresolved issue is whether GluA2-lacking AMPARs in the striatum participate in strategy switching as a molecular event downstream of NMDAR activation.

The present study clarified whether NMDAR and AMPAR activation in the striatum is necessary for cognitive flexibility. We compared the total numbers of trials and errors to reach criterion between control and antagonist-treated groups during set-shifting and reversal learning tasks. Rats were microinjected with the NMDAR-selective antagonist (2*R*)-amino-5-phosphonopentanoate (AP5) or GluA2-lacking AMPAR antagonist 1-naphthylacetylspermine (NASPM) into subregions of the striatum before performing the behavioral tasks. Four subregions of the striatum are the nucleus accumbens (NAc) core, NAc shell, DMStr, and DLStr. Traditionally, the term “striatum” refers to the dorsal striatum. The NAc is referred to as the ventral striatum because it is considered to be the predominant part of the ventral striatum. The shell and core subregions of the NAc have different connectivity, with the shell more closely related to the limbic system and the core more closely related to the dorsal striatum (Berendse and Groenewegen, 1990; Voorn et al., 2004). In the present study, the types of errors were further analyzed to reveal possible behavioral components that were affected by the treatments, including prior memory and the learning and maintenance of new strategies.

## Materials and methods

### Subjects

Male Sprague-Dawley rats (Vital River Animal Center, Beijing, China), weighing 280–300 g upon arrival, were used in the study. The rats were individually housed in stainless-steel sliding-drawer-type cages (50 cm length × 32 cm width × 22 cm height) with a wire mesh floor and pine wood-shavings below the cages. The rats were maintained on a 12/12 h light/dark cycle (lights on at 7:00 AM), with water and food available *ad libitum*. All of the rats were gently handled daily for 1 week before the behavioral experiment began. The experimental procedures were approved by the International Review Board of the Institute of Psychology, Chinese Academy of Sciences, and were in compliance with the National Institutes of Health Guide for Care and Use of Laboratory Animals.

### Apparatus

The behavioral experiments were conducted in eight operant chambers (29 × 29 × 26 cm; Anilab Software and Instruments Co., Ltd., Ningbo, China) enclosed in sound-attenuating boxes. Each chamber was fitted with two nosepoke operandi, one of which was located on each side of a central liquid receptacle. Two yellow light-emitting-diode cue lights (20 mW) were separately situated inside the nosepoke holes. A white chamber light was located 20 cm above the right nosepoke hole. Solutions were delivered through a metal spout that was attached to a 60-ml syringe pump with tubing that delivered fluid at a rate of 34.450 ml/min. The pumps were calibrated to dispense 0.08 ml (0.139 s) of solution into a liquid receptacle for each reinforcement. A timeout period of 10 s followed each reinforcement, during which further nosepokes produced no effect.

### Surgery

The rats were anesthetized with sodium pentobarbital (60 mg/kg, i.p.) before cannula implantation surgery. After being placed in a stereotaxic frame, a midsagittal incision was made to retract the scalp and expose the cranium. Bilateral 23-gauge stainless-steel guide cannulas (Plastics One, Roanoke, VA, USA) were implanted into the NAc core (flat skull: anterior/posterior [AP], +1.6 mm; medial/lateral [ML], ±1.5 mm from bregma; dorsal/ventral [DV], −7.2 mm from dura), NAc shell (AP, +1.7 mm; ML, ±1.0 mm; DV, −7.8 mm], DMStr (AP, +1.0 mm; ML, ±1.8 mm; DV, −4.5 mm), or DLStr (AP, +1.2 mm; ML, ±3.5 mm; DV, −4.5 mm). The coordinates were based on the Paxinos and Watson (1997) rat brain atlas. Four jeweler’s screws and dental acrylic were used to secure the cannulae to the skull. Thirty-gauge obturators were placed on the ends of the guide cannulae until the injections were made. Each rat was given at least 7 days to recover from surgery before behavioral training.

### Behavioral flexibility test

#### Habituation

At the beginning of habituation, the rats were placed in the operant chambers. They were allowed to freely explore the chamber for 15 min with the nosepoke light off. Sucrose reinforcement was unavailable during this period. A 60-min session was used to train the rats to learn that the sucrose solution was available in the central liquid receptacle. The chamber was illuminated by two nosepoke lights, and liquid was delivered into the central liquid receptacle on a variable interval (VI) 40-s schedule (Chudasama and Robbins, 2003). One second before the liquid was dispensed, the house light (as the conditioned stimulus) was turned on and remained illuminated for 4 s. During habituation, 20% sucrose (wt/vol) in tap water was used as the liquid reward. To ensure that the rats drank more sucrose in the operant chambers, water and food were removed from their home cages 2 and 18 h, respectively, before habituation began. Throughout operant training and testing, the rats were allowed access to food for 1 h per day after each daily session to maintain their body weight above 85% of their baseline weight (Dias-Ferreira et al., 2009).

Side preference was assessed during the habituation period. In this session, the rats could nosepoke either the left or right hole, which were not associated with sucrose reward. To balance the effect of side preference, the number of nosepokes was recorded. If the number of left or right nosepokes was two-times higher than the number of nosepokes on the other side, then the former was designated the preferred side.

#### Visual-cue learning

Twenty-four hours after the habituation session, the rats were required to respond at the nosepoke hole with an illuminated visual-cue stimulus light. During illumination of the active nosepoke hole light, a nosepoke into the correct hole (active hole) turned on the house light and switched on the syringe pump 1 s later. Nosepokes in the other hole (inactive hole) had no scheduled consequences. The acquisition criterion was defined as nine consecutive correct responses. A similar criterion was used in previous studies (Floresco et al., 2008; Haluk and Floresco, 2009; Dalton et al., 2011). The rats were returned to their home cages if they did not successfully achieve the acquisition criterion after 100 trials. Two hours later, the rats were removed to the operant chamber, and a new session began until the criterion of nine consecutive correct responses was reached.

#### Set-shifting

One day after visual-cue learning, the set-shifting task was conducted using the same group of rats. The acquisition of this discrimination task required the animal to cease the use of the previous visual-cue discrimination strategy and instead use a direction-discrimination strategy to obtain the sucrose reward. A correct response consisted of responding at the nosepoke hole that was opposite to the rat’s side bias (left or right), regardless of the location of the stimulus light that was illuminated previously in one of the nosepoke holes. Similar to the initial visual-cue discrimination, in every pair of trials, the left or right stimulus light was illuminated once, and the order was random within the pair of trials. The trials were conducted identically to the initial visual-cue discrimination trials. In each trial, the nosepoke hole that the animal chose and the location of the stimulus light were recorded. The trials continued until either (1) the rat achieved criterion performance of nine consecutive correct responses or (2) 100 trials were completed. In this experiment, all of the rats achieved criterion performance within this allotted number of trials.

#### Reversal learning

One day after the set-shifting trials, the rats were required to learn a reversal of the response rule. The rats had to nosepoke in the hole opposite to the one they learned during the set-shifting task on the preceding day. Thus, if a rat was required to nosepoke in the left hole during the set-shifting task, then it had to nosepoke in the right hole in the reversal learning task, regardless of the position of the visual cue. The other aspects of the training were identical to the response discrimination training day. One of the visual-cue lights was illuminated prior to the start of each trial and served as a distracting stimulus.

#### Error analysis

We performed an error analysis of the set-shifting and reversal-learning data as reported previously (Ragozzino and Choi, 2004; Floresco et al., 2008; Ghods-Sharifi et al., 2008). A perseverative error occurred when a rat responded in a nosepoke hole while the stimulus light was illuminated during trials in which the rat was required to nosepoke in the opposite hole. Eight of every 16 consecutive trials required the rat to respond in this manner. These types of trials were separated into consecutive blocks with four trials each. “Perseverative errors” were scored when a rat made such errors in three or more trials per block of four trials that required a nosepoke in the hole that did not have the stimulus light illuminated. When a rat made two or fewer perseverative errors in a block for the first time, all subsequent errors of this type were no longer counted as perseverative errors because at this point the rat was using the original strategy less than 75% of the time (as was used for the operant chamber-based procedures). Instead, these errors were now scored as “regressive errors”. “Never-reinforced errors” were scored when a rat nosepoked in the incorrect hole in trials in which the visual-cue light was illuminated at the same hole that the rat was required to nosepoke during the set-shifting task. During the reversal learning session, errors were scored as nosepokes in the hole that was rewarded during the set-shifting task and were subdivided into perseverative and regressive errors using a procedure that was similar to the one used to analyze these types of errors during the set-shifting task. Perseverative errors were scored when a rat made three or more perseverative within a block of four trials, excluding the first block. When a rat made fewer than three errors within a block of four trials, all subsequent errors were scored as regressive errors.

### Microinfusion procedure

Before testing set-shifting, reversal learning, or initial visual-cue learning, the rats received a bilateral intra-brain region injection of NASPM (1.25 μg per hemisphere, 5 μg/μl), AP5 (0.25 μg per hemisphere, 0.5 μg/μl), or their corresponding vehicle solution (0.9% NaCl solution). Saline or the antagonist was infused at a rate of 0.25 μl/100 s (NASPM) or 0.5 μl/100 s (AP5) by a microsyringe pump. The injection needles were left in place for an additional 1 min to allow for drug diffusion. Following the infusion, the rats were placed in the operant chamber. Behavioral testing commenced 5 min (NASPM) or 15 min (AP5) later. NASPM and AP5 were purchased from Sigma.

### Histology

**Figure 1:**
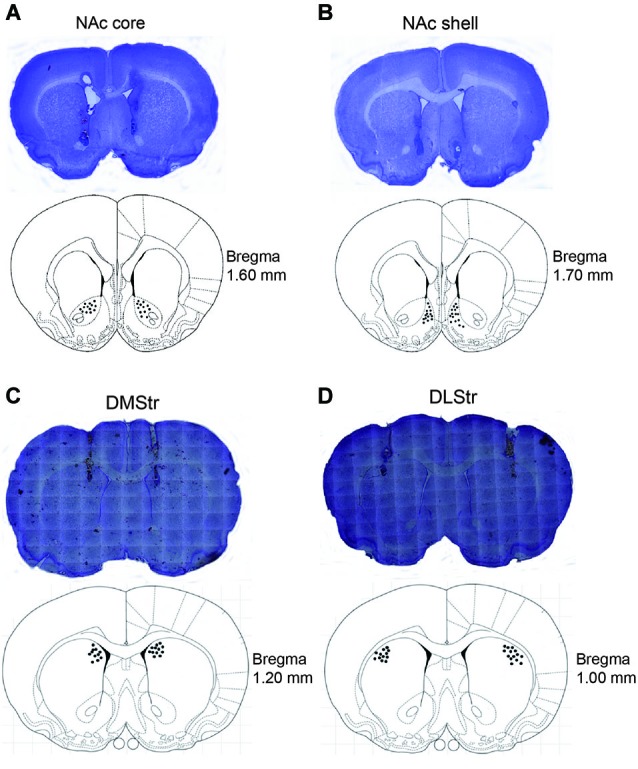
**Images showing cannula tracks and needle-tip locations in the NAc core (A), NAc shell (B), DMStr (C), and DLStr (D)**. After the behavioral tests, the rats were sacrificed. Brains were removed and fixed in a 4% formalin solution. The brain slices were collected in 50 μm coronal sections prior to being mounted and stained with Nissl staining solution. Images are shown on the top of each pattern. The distribution of the infusion sites was plotted on drawings of coronal sections from the Paxinos and Watson (1997) atlas. NAc, nucleus accumbens; DMStr, dorsomedial striatum; DLStr, dorsolateral striatum.

After behavioral testing, the rats were sacrificed in a carbon dioxide chamber. The brains were removed, fixed in a 4% formalin solution, frozen, sliced into 50 μm coronal sections, mounted on slides, and stained with Cresyl violet. The needle placements were verified with reference to the neuroanatomical Paxinos and Watson (1997) rat brain atlas. Figure 1 shows the approximate points of the bilateral infusions into the NAc core, NAc shell, DMStr, and DLStr. Data from rats with improper cannula placements were excluded from the analyses (9% of the total number of animals).

### Data analysis

The data were analyzed using GraphPad Prism 6.0 software. The numbers of trials and errors to reach criterion were separately analyzed using two-way analysis of variance (ANOVA) for each brain region, with Treatment as the within-subjects factor and Training phase as the between-subjects factor. Significant main effects in the ANOVA were followed by Bonferroni multiple-comparison *post hoc* tests. Subsequent targeted analyses that compared the numbers of each type of error were conducted using two-way ANOVAs. The effects of AP5 on simple learning were analyzed using two-tailed *t*-tests per NAc subregion. The criterion for statistical significance was set to *p* < 0.05.

## Results

### Effects of blocking NMDARs in NAc shell and core on set-shifting and reversal learning

**Figure 2:**
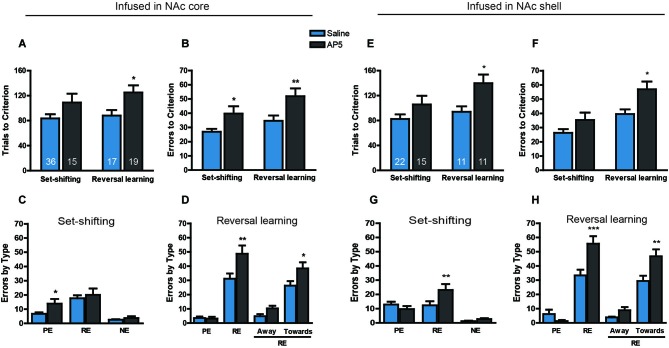
**Effects of AP5 infused into the NAc core and NAc shell on behavioral flexibility**. The behavioral procedure included three phases: cue learning, set-shifting, and reversal learning. Set-shifting and reversal learning were tested 24 and 48 h, respectively, after cue learning. Each rat was infused with 0.5 μg (0.5 μl) AP5 (dissolved in saline) per side before the test. The sample size in each group is shown in the corresponding column. **(A)** Number of trials and **(B)** number of errors to reach criterion in the set-shifting and reversal learning tasks following intra-NAc core infusions. **(C,D)** Analysis of the types of errors during set-shifting **(C)** and reversal learning **(D)** in rats that received AP5 treatment in the NAc core. **(E)** Number of trials and **(F)** number of errors to reach criterion in the set-shifting and reversal learning tasks following intra-NAc shell infusions. **(G,H)** Analysis of the types of errors during set-shifting **(G)** and reversal learning **(H)** in rats that received AP5 treatment in the NAc shell. * *p* < 0.05, ** *p* < 0.01, *** *p* < 0.001. PE, perseverative error; RE, regressive error; NE, non-reinforced error. During the reversal learning task, regressive errors were further divided into “away” and “toward” the visual cue.

Fifty-three rats were trained to obtain 20% sucrose solution by nosepoking the hole associated with the visual cue. Twenty-four hours later, 15 rats were infused with AP5 into the NAc core before the set-shifting task, and 36 rats were infused with the corresponding saline vehicle. Twenty-four hours after the set-shifting task, the rats that received saline treatment during the set-shifting task were divided into an AP5 infusion group (19 rats) and saline infusion group (17 rats) for the reversal learning task. The two-way ANOVA of these data revealed a significant effect of Treatment on both the number of trials to reach criterion (*F*_(1,83)_ = 9.441, *p* < 0.01; Figure 2A) and the number of errors before reaching the criterion (*F*_(1,83)_ = 14.54, *p* < 0.001; Figure 2B). Multiple-comparison analysis revealed that the rats that received AP5 infusion required significantly more trials (*p* < 0.05) and made more errors (*p* < 0.01) compared with saline infusion during reversal learning. AP5 infusion significantly increased the number of errors to reach criterion (*p* < 0.05) but not the number of trials to reach criterion (*p* > 0.05) during the set-shifting task. Subsequent analysis of the types of errors during the set-shifting task made by the rats that received AP5 revealed a significant effect of Treatment (*F*_(1,147)_ = 4.392, *p* < 0.05; Figure 2C). Multiple-comparison analysis indicated that this treatment increased the number of perseverative errors (*p* < 0.05) but not regressive errors or never-reinforced errors (*p* > 0.05). In the reversal learning task, the analysis of the types of errors also showed a significant effect of Treatment (*F*_(1,68)_ = 5.624, *p* < 0.05; Figure 2D). However, this difference resulted from an AP5-induced increase in the number of regressive errors (*p* < 0.05) and not perseverative errors (*p* > 0.05). Two subtypes of regressive errors were further analyzed by two-way ANOVA. The results showed a significance increase in the number of regressive errors toward the visual cue (*p* < 0.05) and a trend toward an increase in the number of regressive errors away from the visual cue.

The effects of AP5 infusion in the NAc shell on behavioral flexibility are shown in Figure 2. A total of 37 rats were assigned to either the saline or drug treatment group based on their performance during the visual-cue training. AP5 was infused into the NAc shell before the set-shifting task, 24 h after initial cue learning. Twenty-four hours later, half of the rats were infused with saline vehicle during the set-shifting task and infused with AP5 before the reversal learning task. The other half were administered saline. The analysis of these data revealed a significant effect of Treatment on the number of both trials (*F*_(1,55)_ = 9.426, *p* < 0.01; Figure 2E) and errors (*F*_(1,55)_ = 9.96, *p* < 0.01; Figure 2F) to reach criterion. Multiple-comparison analysis revealed that infusions of the NMDAR antagonist AP5 induced a weak effect on performance during the set-shifting task, whereas AP5 infusion produced a pronounced impairment in reversal learning, leading to a significant increase in the number of trials and errors to reach criterion (both *p* < 0.05). Subsequent targeted analysis of the types of errors made during the set-shifting task after AP5 infusion revealed a significant effect of Treatment (*F*_(1,105)_ = 2.494, *p* < 0.05; Figure 2G). Infusions of AP5 into the NAc shell increased the number of regressive errors (*p* < 0.05), without affecting perseverative or never-reinforced errors (*p* > 0.05). In the reversal learning task, the analysis of the types of errors also revealed a significant effect of Treatment (*F*_(1,40)_ = 5.662, *p* < 0.05; Figure 2H), with a robust increase in the number of regressive errors (*p* < 0.001). Two subtypes of regressive errors were analyzed by two-way ANOVA. The results showed a significance increase in the number of regressive errors toward the visual cue (*p* < 0.05) and a trend toward an increase in the number of regressive errors away from the visual cue.

**Figure 3:**
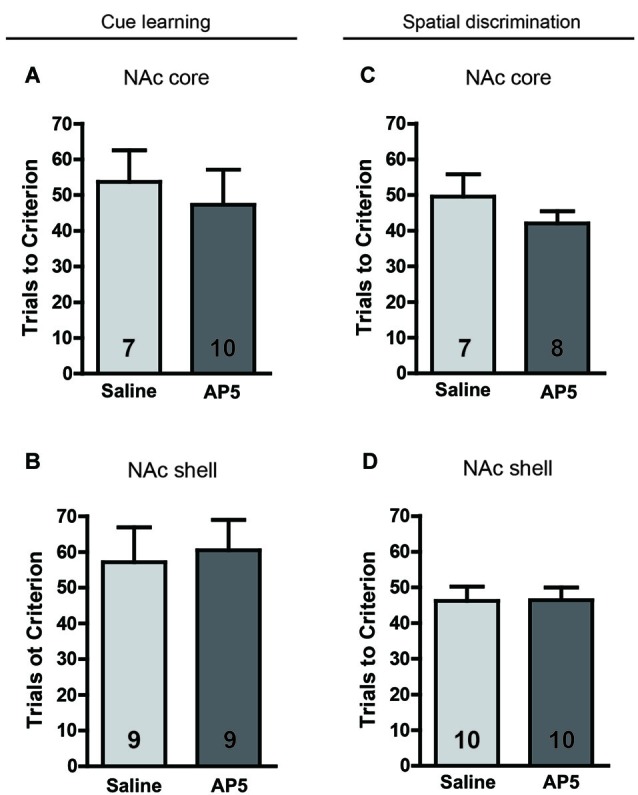
**No effect of AP5 infused into the NAc core or shell on initial visual-cue learning (A, NAc core; B, NAc shell) or spatial discrimination (C, NAc core; D, NAc shell)**. In this experiment, only association learning tasks were conducted. The rats were required to learn an association between a visual cue and reinforcement or between a spatial response and reinforcement. Before the behavioral test, 0.5 μg (0.5 μl) of an AP5 solution or saline vehicle was infused into the NAc core or shell per side. The sample size in each group is shown in the corresponding column.

To assess whether the blockade of NMDARs in the NAc affects the initial learning associated with visual-cue and spatial discrimination, we infused AP5 into the NAc core and shell, respectively, before initial training. The data were analyzed using separate *t*-tests for each kind of learning task based on each brain region. Figure 3 shows that AP5 infusion in the NAc core or shell did not alter simple learning performance, regardless of cue-associated or spatial discrimination (all *t* < 1.1, no significant differences).

### Effects of blocking NMDARs in the DMStr and DLStr on set-shifting and reversal learning

**Figure 4:**
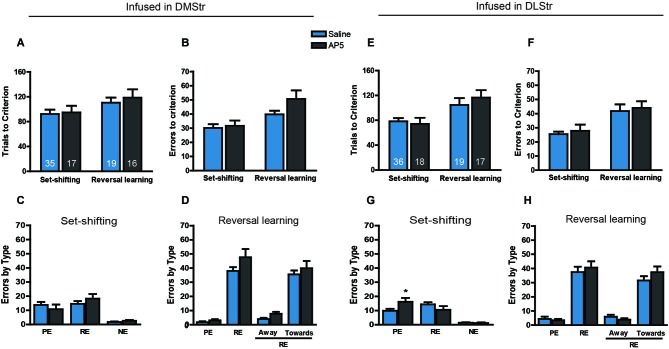
**Effects of AP5 infused into dorsomedial striatum and dorsolateral striatum on behavioral flexibility**. The behavioral procedure included three phases: cue learning, set-shifting, and reversal learning. Set-shifting and reversal learning were tested 24 and 48 h, respectively, after cue learning. Each rat was infused with 0.5 μg (0.5 μl) AP5 (dissolved in saline) per side before the test. The sample size in each group is shown in the corresponding column. **(A)** Number of trials and **(B)** number of errors to reach criterion in the set-shifting and reversal learning tasks following intra-DMStr infusions. **(C,D)** Analysis of the types of errors during set-shifting **(C)** and reversal learning **(D)** in rats that received AP5 treatment in the DMStr. **(E)** Number of trials and **(F)** number of errors to reach criterion in the set-shifting and reversal learning tasks following intra-DLStr infusions. **(G,H)** Analysis of the types of errors during set-shifting **(G)** and reversal learning **(H)** in rats that received AP5 treatment in the DLStr. * *p* < 0.05. PE, perseverative error; RE, regressive error; NE, non-reinforced error. During the reversal learning task, regressive errors were further divided into “away” and “toward” the visual cue.

The effects of infusions of saline and AP5 into the DMStr and DLStr on set-shifting and reversal learning are presented in Figure 4. The experimental procedures were the same as those used in the NAc treatment experiments. After initial training, the set-shifting and reversal learning tasks were performed on the second and third days, respectively. AP5 was infused into the corresponding brain region before the behavioral tests. Rats that received infusions of the NMDAR antagonist AP5 in the DMStr required a comparable number of trials (*F*_(1,83)_ = 0.3002, *p* > 0.05; Figure 4A) and made a comparable number of errors (*F*_(1,83)_ = 2.7956, *p* > 0.05; Figure 4B) to reach criterion compared with controls. A nonsignificant trend toward an increase was observed with AP infusions during the reversal learning task, suggesting that the blockade of NMDARs in the DMStr mildly impaired intradimensional strategy-shifting ability without disrupting extradimentional strategy-switching. The analysis of the types of errors revealed no significant difference between groups in set-shifting (*F*_(1,66)_ = 0.0822, *p* > 0.05; Figure 4C) or reversal learning (*F*_(1,66)_ = 0.1698, *p* > 0.05; Figure 4D).

Similarly, no difference was found between the AP5-treated and control groups in the number of trials (*F*_(1,86)_ = 0.6595, *p* > 0.05; Figure 4E) or errors (*F*_(1,86)_ = 0.5259, *p* > 0.05; Figure 4F) to reach criterion. The analysis of the error data indicated that NMDAR blockade induced a statistically significant Treatment × Error type interaction in set-shifting (*F*_(2,156)_ = 0.0089, *p* < 0.01; Figure 4G). Infusions of AP5 induced a pronounced increase in the number of perseverative errors (*p* < 0.05), without affecting regressive or never-reinforced errors.

### Effects of blocking GluA2-lacking AMPARs in the striatum on set-shifting and reversal learning

**Figure 5:**
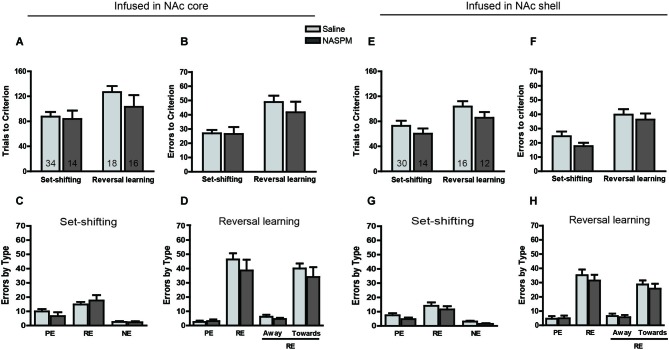
**Effects of NASPM infused into the NAc core and NAc shell on behavioral flexibility**. The behavioral procedure included three phases: cue learning, set-shifting, and reversal learning. Set-shifting and reversal learning were tested 24 and 48 h, respectively, after cue learning. Each rat was infused with 1.25 μg (0.25 μl) NASPM (dissolved in saline) per side before the test. The sample size in each group is shown in the corresponding column. **(A)** Number of trials and **(B)** number of errors to reach criterion in the set-shifting and reversal learning tasks following intra-NAc core infusions. **(C,D)** Analysis of the types of errors during set-shifting **(C)** and reversal learning **(D)** in rats that received NASPM treatment in the NAc core. **(E)** Number of trials and **(F)** number of errors to reach criterion in the set-shifting and reversal learning tasks following intra-NAc shell infusions. **(G,H)** Analysis of the types of errors during set-shifting **(G)** and reversal learning **(H)** in rats that received NASPM treatment in the NAc shell. No changes were found in the set-shifting or reversal learning phase. PE, perseverative error; RE, regressive error; NE, non-reinforced error. During the reversal learning task, regressive errors were further divided into “away” and “toward” the visual cue.

**Figure 6:**
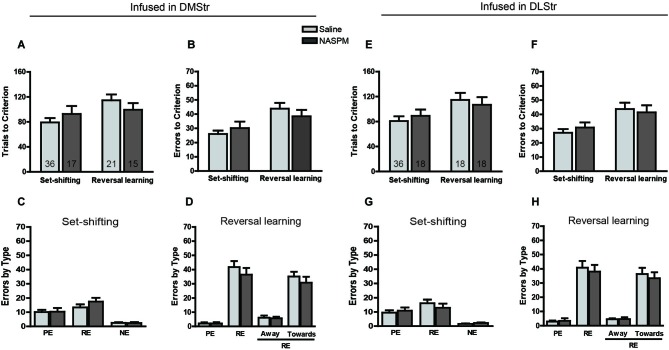
**Effects of NASPM infused into the dorsomedial striatum and dorsolateral striatum on behavioral flexibility**. The behavioral procedure included three phases: cue learning, set-shifting, and reversal learning. Set-shifting and reversal learning were tested 24 and 48 h, respectively, after cue learning. Each rat was infused with 1.25 μg (0.25 μl) NASPM (dissolved in saline) per side before the test. The sample size in each group is shown in the corresponding column. **(A)** Number of trials and **(B)** number of errors to reach criterion in the set-shifting and reversal learning tasks following intra-DMStr infusions. **(C,D)** Analysis of the types of errors during set-shifting **(C)** and reversal learning **(D)** in rats that received NASPM treatment in the DMStr. **(E)** Number of trials and **(F)** number of errors to reach criterion in the set-shifting and reversal learning tasks following intra-DLStr infusions. **(G,H)** Analysis of the types of errors during set-shifting **(G)** and reversal learning **(H)** in rats that received NASPM treatment in the DLStr. No changes were found in the set-shifting or reversal learning phases. PE, perseverative error; RE, regressive error; NE, non-reinforced error. During the reversal learning task, regressive errors were further divided into “away” and “toward” the visual cue.

In this experimental session, NASPM was used to determine whether the blockade of GluA2-lacking AMPARs in different striatal subregions induces different strategy switching results. Twenty-four hours after initially learning the association between the visual cue and sucrose reinforcement, the set-shifting task was performed. The rats were required to learn a novel strategy to obtain the reward (i.e., by always nosepoking the left or right hole). The NASPM solution was infused into the NAc core, NAc shell, DMStr, or DLStr 5 min before testing in separate experiments. Twenty-four hours after the set-shifting task, half of the rats, which were infused with saline during the set-shifting task, were infused with NASPM solution before the reversal learning task. The other half of the rats were administered saline as a control. The results are presented in Figures 5, 6, showing the number of trials to reach criterion and the types of errors. Infusions of NASPM in any striatal subregion did not significantly affect the number of trials to reach criterion (NAc core: *F*_(1,78)_ = 0.2662, *p* > 0.05, Figure 5A; NAc shell: *F*_(1,68)_ = 2.64, *p* > 0.05, Figure 5E; DMStr: *F*_(1,85)_ = 0.0064, *p* > 0.05, Figure 6A; DLStr: *F*_(1,86)_ = 0.0005, *p* > 0.05, Figure 6E). Similarly, NASPM infusion in any of the striatal subregions did not induce any changes in the number of errors to reach criterion (all *F* < 1.9, no significant differences). A subsequent analysis of the types of errors made during the set-shifting and reversal learning tasks revealed no difference between the NASPM- and saline-infused groups. These data indicate that Ca^2+^-permeable AMPARs in the striatum do not play a crucial role in behavioral flexibility. The dose of NASPM used in the present study has been shown previously to affect reward-associated behavior (Carr et al., 2009). We may need to test doses of NASPM higher than 2.5 μg in our future studies, but such doses may carry the risk of affecting the reward system.

## Discussion

The present study revealed that the blockade of NMDARs in the NAc core impaired set-shifting performance by increasing perseverative errors. The suppression of NMDAR-mediated neurotransmission in the NAc shell induced a deficit in set-shifting by enhancing regressive errors. These findings indicate that the activation of NMDARs in the NAc core alters prior learning or inhibits responses that are no longer appropriate, whereas NMDARs in the NAc shell play an important role in learning and maintaining novel strategies. Reversal learning reflects intradimensional strategy switching and was tested 24 h after the set-shifting task. AP5 infusion in the NAc shell and core significantly increased the total number of trials to reach criterion and the number of regressive errors, indicating that AP5-treated rats exhibited dysfunction in novel strategy learning or maintenance. In contrast, no significant alteration was induced by AP5 infusion into the DMStr or DLStr in the set-shifting or reversal learning task. We also tested the selective GluA2-lacking AMPAR antagonist NASPM to assess the effects of blocking GluA2-lacking AMPARs in different striatal subregions on behavioral flexibility. No effect was found during the set-shifting or reversal learning task.

Schizophrenia patients exhibit impaired cognitive flexibility measured by the Wisconsin Card Sorting Test (WCST; Everett et al., 2001). In the extradimensional shift stage, both schizophrenia patients and frontal-lesioned subjects exhibit pronounced deficits, whereas only schizophrenia patients make significantly more errors in the intradimensional reversal stage (Pantelis et al., 1999). This suggests that other brain regions, in addition to the frontal cortex, are abnormal in schizophrenia patients. The striatum may be an important target among these regions. Subjects with schizophrenia exhibit striatal dysfunction during reward-related reversal learning (Schlagenhauf et al., 2014), and the striatum receives excitatory projections from the frontal cortex (Reynolds and Zahm, 2005; Xu and Südhof, 2013).

By microinjecting γ-aminobutyric acid receptor agonists to inactivate the NAc, Floresco et al. (2006) demonstrated dissociable contributions made by neurons in the NAc core and shell to behavioral flexibility during the set shifting task. These authors found that set-shifting was impaired after inactivation of the NAc core but not shell, attributable to disruption of the acquisition and maintenance of a new strategy. However, we only blocked NMDAR-mediated transmission, which may be a major reason for the discrepant results between the study by Floresco et al. and the present study. In addition to receptor targets, the discrepancies between these two studies may be attributable to differences in the training protocols. An automated procedure was used in the present study, which decreases the likelihood of experimental bias and increases consistency in timing and stimulus intensity compared with the cross-maze setup that was utilized by Floresco et al. The NAc core receives projections from the prelimbic cortex (PL), whereas the NAc shell receives projections from the infralimbic cortex (IL; Voorn et al., 2004). Lesions of the PL impaired set-shifting performance by inducing deficits in inhibiting the relevant learned strategy. The IL may support behavioral flexibility by maintaining the reliable execution of a new choice pattern (Birrell and Brown, 2000; Ragozzino, 2007). In contrast to the differential roles of NMDAR activation between NAc subregions in extradimensional strategy shifting, NMDARs in both the NAc shell and core make the same contribution to intradimensional strategy switching. The activation of NMDARs in the NAc may facilitate the learning and maintenance of new strategies. In the present study, the rats that underwent reversal learning testing were previously subjected to the set-shifting task. However, the impaired ability in spatial reversal induced by NMDAR blockade did not result from alterations in set-shifting because no interaction was found between “away” and “toward” visual-cue regressive errors. An impaired ability in reversal learning has been previously reported in studies in which the ventral striatum and dorsal striatum were subjected to excitotoxic lesions (Ferry et al., 2000). Similar deficits are also found with lesions and inactivation of the OFC (Chudasama and Robbins, 2003; Boulougouris et al., 2007; Ghods-Sharifi et al., 2008). An interaction has been suggested to exist between striatum and OFC functions in reversal learning (Frank and Claus, 2006). However, the modulation of reversal learning ability by the OFC depends on task difficulty. When the reversal task was performed between two strategies, inactivation or lesions of the OFC produced perseveration (Bohn et al., 2003; Chudasama and Robbins, 2003; Boulougouris et al., 2007). In contrast, OFC inactivation increased both perseverative and regressive errors when four-choice odor discrimination was required (Ragozzino, 2007). In the present study, reversal learning based on an operant task may be more difficult than two-choice patterns. Thus, the blockade of NMDARs in the NAc increased regressive errors in the present study. Although an impaired ability in working memory has been demonstrated in some cases of cognitive inflexibility (Blot et al., 2013; Darvas et al., 2014), working memory performance in the present study did not appear to be altered by blocking NMDARs in the NAc. No significant changes were found in the cue-learning task or spatial response when AP5 was microinjected into the NAc shell or core.

The dorsal striatum contributes less to flexible behaviors compared with the ventral striatum, although impaired reversal learning performance suggests that NMDAR-mediated glutamate transmission in the DMStr plays a role in behavioral flexibility by at least partially supporting a shift in intradimensional strategies. Similar results have been reported in previous studies that used a reversal learning task (Palencia and Ragozzino, 2004, 2005). However, inconsistent results were found in the set-shifting task with DMStr treatment between the present study and previous reports. We did not observe any apparent change in the set-shifting task when NMDAR activation was blocked in the DMStr. Ragozzino et al. inactivated the DMStr based on a cross-maze experimental design and found that this region mediates the maintenance of novel strategies after perseveration ceases (Ragozzino et al., 2002). In the DLStr, the increase in the number of perseverative errors in the set-shifting task may be attributable to the modulatory role of this region in stimulus-response habits (Jog et al., 1999).

NMDAR-dependent long-term depression (LTD) and synaptic changes can be modulated by endocytosis of GluA2-containing AMPARs (Lim et al., 2012), suggesting that GluA2-lacking AMPARs may replace GluA2-containing AMPARs during learning. However, no deficit was observed after NASPM treatment, indicating that the activation of GluA2-lacking AMPARs is not a downstream event of NMDAR-induced transmission involved in flexible behaviors. AMPAR antagonism with LY293558 in the mPFC induced general cognitive deficits (Stefani et al., 2003), and infusions of the AMPAR antagonist DNQX into the ventral striatum impaired spatial information processing in a nonassociative task (Roullet et al., 2001). These previous studies suggest that AMPARs may be involved in simple learning and general cognition rather than specifically cognitive execution. Under physiological conditions, most AMPARs in the striatum are either GluA1/2 or GluA2/3, whereas <10% are homomeric GluA1 or GluA1/3 (Boudreau et al., 2007). Furthermore, although the endocytosis of GluA2-containing AMPARs is a mechanism associated with synaptic long-term plasticity during learning (Liu and Zukin, 2007), other forms of plasticity may also be involved in this associative task, such as mGluR-dependent patterns (Anwyl, 2009).

In summary, pharmacological tools associated with glutamate transmission may provide a potential clinical treatment for schizophrenia. In addition to the frontal cortex, the ventral striatum may be another important brain region for adjusting behavioral flexibility. These findings suggest that NMDAR-mediated glutamate transmission in the ventral striatum makes more of a contribution to cognitive execution than the dorsal striatum.

## Author contributions

Xuekun Ding, Yanhua Qiao, and Chenji Piao performed experiments, analyzed data and wrote the manuscript. Xigeng Zheng and Zhengkui Liu managed the literature searches and analyses. Jing Liang designed the study, wrote the protocol and finalized the manuscript. All authors contributed to and have approved the final manuscript and agreed to be accountable for all aspects of the work.

## Conflict of interest statement

The authors declare that the research was conducted in the absence of any commercial or financial relationships that could be construed as a potential conflict of interest.

## Abbreviations

DMStr: Dorsomedial striatum
DLStr: Dorsolateral striatum
NASPM: 1-naphthylacetylspermine
PE: Perseverative error
RE: Regressive error
NE: Non-reinforced error.

## References

[B1] Ai H. Yang W. Ye M. Lu W. Yao L. Luo J. H. (2011). Differential regulation of AMPA receptor GluA1 phosphorylation at serine 831 and 845 associated with activation of NMDA receptor subpopulations. Neurosci. Lett. 497, 94–98 10.1016/j.neulet.2011.04.038 21539895

[B2] Anwyl R. (2009). Metabotropic glutamate receptor-dependent long-term potentiation. Neuropharmacology 56, 735–740 10.1016/j.neuropharm.2009.01.002 19705571

[B3] Berendse H. W. Groenewegen H. J. (1990). Organization of the thalamostriatal projections in the rat, with special emphasis on the ventral striatum. J. Comp. Neurol. 299, 187–228 10.1002/cne.902990206 2172326

[B4] Birrell J. M. Brown V. J. (2000). Medial frontal cortex mediates perceptual attentional set shifting in the rat. J. Neurosci. 20, 4320–4324 10818167 10.1523/JNEUROSCI.20-11-04320.2000 PMC6772641

[B5] Blot K. Kimura S. I. Bai J. Kemp A. Manahan-Vaughan D. Giros B. (2013). Modulation of hippocampus-prefrontal cortex synaptic transmission and disruption of executive cognitive functions by MK-801. Cereb. Cortex [Epub ahead of print]. 10.1093/cercor/bht329 24304584

[B6] Bohn I. Giertler C. Hauber W. (2003). Orbital prefrontal cortex and guidance of instrumental behaviour in rats under reversal conditions. Behav. Brain Res. 143, 49–56 10.1016/s0166-4328(03)00008-1 12842295

[B7] Boudreau A. C. Reimers J. M. Milovanovic M. Wolf M. E. (2007). Cell surface AMPA receptors in the rat nucleus accumbens increase during cocaine withdrawal but internalize after cocaine challenge in association with altered activation of mitogen-activated protein kinases. J. Neurosci. 27, 10621–10635 10.1523/jneurosci.2163-07.2007 17898233 PMC2856315

[B8] Boulougouris V. Dalley J. W. Robbins T. W. (2007). Effects of orbitofrontal, infralimbic and prelimbic cortical lesions on serial spatial reversal learning in the rat. Behav. Brain Res. 179, 219–228 10.1016/j.bbr.2007.02.005 17337305

[B9] Carr K. D. Cabeza de Vaca S. Sun Y. Chau L. S. (2009). Reward-potentiating effects of D-1 dopamine receptor agonist and AMPAR GluR1 antagonist in nucleus accumbens shell and their modulation by food restriction. Psychoparmacology (Berl) 202, 731–743 10.1007/s00213-008-1355-9 18841347 PMC2805715

[B10] Chamberlain S. R. Menzies L. Hampshire A. Suckling J. Fineberg N. A. del Campo N. (2008). Orbitofrontal dysfunction in patients with obsessive-compulsive disorder and their unaffected relatives. Science 321, 421–422 10.1126/science.1154433 18635808

[B11] Chudasama Y. Robbins T. W. (2003). Dissociable contributions of the orbitofrontal and infralimbic cortex to pavlovian autoshaping and discrimination reversal learning: further evidence for the functional heterogeneity of the rodent frontal cortex. J. Neurosci. 23, 8771–8780 14507977 10.1523/JNEUROSCI.23-25-08771.2003 PMC6740430

[B12] Dalton G. L. Ma L. M. Phillips A. G. Floresco S. B. (2011). Blockade of NMDA GluN2B receptors selectively impairs behavioral flexibility but not initial discrimination learning. Psychopharmacology (Berl) 216, 525–535 10.1007/s00213-011-2246-z 21384103

[B13] Darvas M. Henschen C. W. Palmiter R. D. (2014). Contributions of signaling by dopamine neurons in dorsal striatum to cognitive behaviors corresponding to those observed in Parkinson’s disease. Neurobiol. Dis. 65, 112–123 10.1016/j.nbd.2014.01.017 24491966 PMC4001780

[B14] Dias-Ferreira E. Sousa J. C. Melo I. Morgado P. Mesquita A. R. Cerqueira J. J. (2009). Chronic stress causes frontostriatal reorganization and affects decision-making. Science 325, 621–625 10.1126/science.1171203 19644122

[B15] Egerton A. Reid L. McGregor S. Cochran S. M. Morris B. J. Pratt J. A. (2008). Subchronic and chronic PCP treatment produces temporally distinct deficits in attentional set shifting and prepulse inhibition in rats. Psychopharmacology (Berl) 198, 37–49 10.1007/s00213-008-1071-5 18427784

[B16] Everett J. Lavoie K. Gagnon J. F. Gosselin N. (2001). Performance of patients with schizophrenia on the Wisconsin Card Sorting Test (WCST). J. Psychiatry Neurosci. 26, 123–130 11291529 PMC1407748

[B17] Ferry A. T. Lu X. C. Price J. L. (2000). Effects of excitotoxic lesions in the ventral striatopallidal—thalamocortical pathway on odor reversal learning: inability to extinguish an incorrect response. Exp. Brain Res. 131, 320–335 10.1007/s002219900240 10789947

[B18] Floresco S. B. Block A. E. Tse M. T. (2008). Inactivation of the medial prefrontal cortex of the rat impairs strategy set-shifting, but not reversal learning, using a novel, automated procedure. Behav. Brain Res. 190, 85–96 10.1016/j.bbr.2008.02.008 18359099

[B19] Floresco S. B. Ghods-Sharifi S. Vexelman C. Magyar O. (2006). Dissociable roles for the nucleus accumbens core and shell in regulating set shifting. J. Neurosci. 26, 2449–2457 10.1523/jneurosci.4431-05.2006 16510723 PMC6793649

[B20] Floresco S. B. Zhang Y. Enomoto T. (2009). Neural circuits subserving behavioral flexibility and their relevance to schizophrenia. Behav. Brain Res. 204, 396–409 10.1016/j.bbr.2008.12.001 19110006

[B21] Frank M. J. Claus E. D. (2006). Anatomy of a decision: striato-orbitofrontal interactions in reinforcement learning, decision making and reversal. Psychol. Rev. 113, 300–326 10.1037/0033-295x.113.2.300 16637763

[B22] Ghods-Sharifi S. Haluk D. M. Floresco S. B. (2008). Differential effects of inactivation of the orbitofrontal cortex on strategy set-shifting and reversal learning. Neurobiol. Learn. Mem. 89, 567–573 10.1016/j.nlm.2007.10.007 18054257

[B23] Haluk D. M. Floresco S. B. (2009). Ventral striatal dopamine modulation of different forms of behavioral flexibility. Neuropsychopharmacology 34, 2041–2052 10.1038/npp.2009.21 19262467

[B24] Hampshire A. Owen A. M. (2006). Fractionating attentional control using event-related fMRI. Cereb. Cortex 16, 1679–1689 10.1093/cercor/bhj116 16436686

[B25] Isaac J. T. Ashby M. C. McBain C. J. (2007). The role of the GluR2 subunit in AMPA receptor function and synaptic plasticity. Neuron 54, 859–871 10.1016/j.neuron.2007.06.001 17582328

[B26] Jaafari N. Henley J. M. Hanley J. G. (2012). PICK1 mediates transient synaptic expression of GluA2-lacking AMPA receptors during glycine-induced AMPA receptor trafficking. J. Neurosci. 32, 11618–11630 10.1523/JNEUROSCI.5068-11.2012 22915106 PMC6703756

[B27] Jentsch J. D. Roth R. H. (1999). The neuropsychopharmacology of phencyclidine: from NMDA receptor hypofunction to the dopamine hypothesis of schizophrenia. Neuropsychopharmacology 20, 201–225 10.1016/s0893-133x(98)00060-8 10063482

[B28] Jog M. S. Kubota Y. Connolly C. I. Hillegaart V. Graybiel A. M. (1999). Building neural representations of habits. Science 286, 1745–1749 10.1126/science.286.5445.1745 10576743

[B29] Lim B. K. Huang K. W. Grueter B. A. Rothwell P. E. Malenka R. C. (2012). Anhedonia requires MC4R-mediated synaptic adaptations in nucleus accumbens. Nature 487, 183–189 10.1038/nature11160 22785313 PMC3397405

[B30] Liu S. J. Zukin R. S. (2007). Ca^2+^-permeable AMPA receptors in synaptic plasticity and neuronal death. Trends Neurosci. 30, 126–134 10.1016/j.tins.2007.01.006 17275103

[B31] Nicolle M. M. Baxter M. G. (2003). Glutamate receptor binding in the frontal cortex and dorsal striatum of aged rats with impaired attentional set-shifting. Eur. J. Neurosci. 18, 3335–3342 10.1111/j.1460-9568.2003.03077.x 14686906

[B32] Opazo P. Labrecque S. Tigaret C. M. Frouin A. Wiseman P. W. De Koninck P. (2010). CaMKII triggers the diffusional trapping of surface AMPARs through phosphorylation of stargazin. Neuron 67, 239–252 10.1016/j.neuron.2010.06.007 20670832

[B33] Orellana G. Slachevsky A. (2013). Executive functioning in schizophrenia. Front. Psychiatry 4:35 10.3389/fpsyt.2013.00035 23805107 PMC3690455

[B34] Palencia C. A. Ragozzino M. E. (2004). The influence of NMDA receptors in the dorsomedial striatum on response reversal learning. Neurobiol. Learn. Mem. 82, 81–89 10.1016/j.nlm.2004.04.004 15341793

[B35] Palencia C. A. Ragozzino M. E. (2005). The contribution of NMDA receptors in the dorsolateral striatum to egocentric response learning. Behav. Neurosci. 119, 953–960 10.1037/0735-7044.119.4.953 16187823

[B36] Pantelis C. Barber F. Z. Barnes T. R. Nelson H. E. Owen A. M. Robbins T. W. (1999). Comparison of set-shifting ability in patients with chronic schizophrenia and frontal lobe damage. Schizophr. Res. 37, 251–270 10.1016/s0920-9964(98)00156-x 10403197

[B37] Paxinos G. Watson C. (1997). The Rat Brain in Stereotaxic Coordinates: Computer Graphics Files. Compact 3rd Edn. San Diego: Academic Press

[B38] Plant K. Pelkey K. A. Bortolotto Z. A. Morita D. Terashima A. McBain C. J. (2006). Transient incorporation of native GluR2-lacking AMPA receptors during hippocampal long-term potentiation. Nat. Neurosci. 9, 602–604 10.1038/nn1678 16582904

[B39] Ragozzino M. E. (2007). The contribution of the medial prefrontal cortex, orbitofrontal cortex and dorsomedial striatum to behavioral flexibility. Ann. N Y Acad. Sci. 1121, 355–375 10.1196/annals.1401.013 17698989

[B40] Ragozzino M. E. Choi D. (2004). Dynamic changes in acetylcholine output in the medial striatum during place reversal learning. Learn. Mem. 11, 70–77 10.1101/lm.65404 14747519 PMC321316

[B41] Ragozzino M. E. Ragozzino K. E. Mizumori S. J. Kesner R. P. (2002). Role of the dorsomedial striatum in behavioral flexibility for response and visual cue discrimination learning. Behav. Neurosci. 116, 105–115 10.1037//0735-7044.116.1.105 11898801 PMC3273324

[B42] Ragozzino M. E. Wilcox C. Raso M. Kesner R. P. (1999). Involvement of rodent prefrontal cortex subregions in strategy switching. Behav. Neurosci. 113, 32–41 10.1037/0735-7044.113.1.32 10197904

[B43] Reynolds S. M. Zahm D. S. (2005). Specificity in the projections of prefrontal and insular cortex to ventral striatopallidum and the extended amygdale. J. Neurosci. 25, 11757–11767 10.1523/jneurosci.3432-05.2005 16354934 PMC6726011

[B44] Roullet P. Sargolini F. Oliverio A. Mele A. (2001). NMDA and AMPA antagonist infusions into the ventral striatum impair different steps of spatial information processing in a nonassociative task in mice. J. Neurosci. 21, 2143–2149 11245698 10.1523/JNEUROSCI.21-06-02143.2001 PMC6762623

[B45] Schlagenhauf F. Huys Q. J. Deserno L. Rapp M. A. Beck A. Heinze H. J. (2014). Striatal dysfunction during reversal learning in unmedicated schizophrenia patients. Neuroimage 89, 171–180 10.1016/j.neuroimage.2013.11.034 24291614 PMC3991847

[B46] Stefani M. R. Groth K. Moghaddam B. (2003). Glutamate receptors in the rat medial prefrontal cortex regulate set-shifting ability. Behav. Neurosci. 117, 728–737 10.1037/0735-7044.117.4.728 12931958

[B47] Toyoda H. Zhao M. G. Ulzhöfer B. Wu L. J. Xu H. Seeburg P. H. (2009). Roles of the AMPA receptor subunit GluA1 but not GluA2 in synaptic potentiation and activation of ERK in the anterior cingulate cortex. Mol. Pain 5:46 10.1186/1744-8069-5-46 19664265 PMC2734546

[B48] Voorn P. Vanderschuren L. J. Groenewegen H. J. Robbins T. W. Pennartz C. M. (2004). Putting a spin on the dorsal-ventral divide of the striatum. Trends Neurosci. 27, 468–474 10.1016/j.tins.2004.06.006 15271494

[B49] Xu W. Südhof T. C. (2013). A neural circuit for memory specificity and generalization. Science 339, 1290–1295 10.1126/science.1229534 23493706 PMC3651700

